# Rules of origin and exporters' value-added^☆^

**DOI:** 10.1016/j.heliyon.2023.e21090

**Published:** 2023-11-14

**Authors:** Dzmitry Kniahin, Marcelo Olarreaga

**Affiliations:** a University of Geneva and International Trade Center, Switzerland; b University of Geneva, CEPR and FERDI, Switzerland

**Keywords:** Rules of origin, Preferential exports

## Abstract

We explore the non-monotonic relationship between the restrictiveness of rules of origin (ROO) and beneficiaries' value-added embedded in preferential exports. Using data for the European Union's GSP schemes, we calculate the value-added maximizing level of ROO restrictiveness. Results suggest that current levels of restrictiveness in the European Union's GSP schemes are not statistically different from optimal levels. More lenient ROO, as sometimes requested by GSP beneficiaries, would reduce their value-added to the benefit of foreign input providers.

## 1 Introduction

Unilateral preferential schemes, such as the Generalized System of Preferences (GSP), grant low-income country exporters preferential market access to high-income countries' markets.^1^ Their objective is to promote beneficiaries' exports and production capacity.^2^ A significant component of preferential schemes are Rules of Origin (ROO), which are used to grant origin to products produced using inputs from different sources, allowing to determine if the final product can be considered as originating in a beneficiary country. ROO ensure that a minimum share of the value of the exported product has been produced in the country to which preferential access has been granted. In the absence of ROO, exports from the rest of the world exporters would be redirected through the preference beneficiary, ultimately eroding any preferential market access granted to the low-income beneficiary and the potential benefits to its productive capacity.^3^

If ROO are set to ensure that the intended beneficiary of a preferential scheme is the one benefiting, they can also erode preferential access by setting ROO that are too restrictive. If to satisfy ROO requirements, exporters are forced to source from high-cost domestic producers instead of low-cost foreign producers, the value of their preferential access declines [16]. A vast literature shows that preference utilization in unilateral schemes is uneven, with exporters in beneficiary countries often preferring to pay the Most Favoured Nation (MFN) tariff rather than satisfy the ROO requirements (see, for example [2,[4], [5], [6],^22^,^23^]).

Thus, whether low-income exporters benefit from GSP preferential schemes granted by high-income countries depends critically on the restrictiveness of ROO. A too-lenient ROO implies that exporters from the rest of the world, rather than the beneficiaries, capture the lion's share of the benefits. In contrast, a too-restrictive ROO implies that nobody benefits from the preferential scheme.

Building on [14], we develop a conceptual framework that shows that beneficiaries' value-added content in preferential exports has a non-monotonic relationship with the level of ROO restrictiveness [14]. called this the ROO Laffer curve. For low levels of ROO restrictiveness, beneficiaries' value-added content in preferential exports increases with the restrictiveness of ROO. Still, for high levels of restrictiveness, the value-added content in preferential exports decreases with the restrictiveness of ROO. Our conceptual framework allows us to derive the level of ROO restrictiveness that maximizes beneficiaries' value-added.

Using a dataset of the EU's GSP tariff preferences, ROO, and preferential exports, we estimate the optimal level of ROO restrictiveness suggested by our empirical model. The empirical results show that the optimal levels of ROO restrictiveness are very close to the actual levels of ROO restrictiveness in the EU's GSP scheme and are not statistically different from them. This implies that a move towards less restrictive ROO will likely result in a decline in the value-added of beneficiaries in preferential exports.

These results are important for at least three reasons.^4^ First, calls for more lenient rules of origin in GSP schemes are common in the academic, policy, and trade negotiation literature. For example [3], argues for a 10% value-added requirement in unilateral preferential schemes, and [9] for their elimination. The current level in the EU's GSP schemes is 46%, and 39% in the case of Least Developed Countries (LDCs). The optimal level we estimate is 41%, indicating that a reduction to 10% would result in a fall in beneficiaries' value-added embedded in preferential exports. Officials from GSP beneficiary countries (particularly the WTO's LDC group) have also often called for more lenient ROO in GSP schemes to promote their economic development (see, for example, the LDC group proposals to WTO members in documents TN/CTD/W/30 in 2006 or TN/AG/GEN/20/Rev.2 in 2011). The latter document calls for adopting value-added criteria in the 15–25% range in LDC preferential schemes. WTO members acknowledged these demands in the Bali Ministerial Declaration in 2013 (WT/MIN(13)/42, WT/L/917). The Nairobi Ministerial Declaration of 2015 agreed that WTO members shall impose value-added requirements below 25% for LDCs (WT/MIN(15)/47). While “shall” is not binding, it indicates a direction to which the Geneva Ministerial Declaration of 2022 has shown commitment (WT/MIN(15)/47, WT/L/917/Add.1). We do not second-guess the good intentions of WTO members and the LDC group in the WTO. However, our results show that the relaxation of ROO's value-added requirements needed to reach the levels recommended by the Nairobi Declaration would most likely result in a decline in LDCs' value-added content in preferential exports.

Second, preferential trade has grown rapidly and is not limited to unilateral schemes. In our sample, total preferential imports of the European Union increased from 60% in 2008 to 78% in 2019. Any preferential scheme is subject to rules of origin; therefore, our paper's conceptual results are relevant to other forms of preferential schemes. Discussions regarding the restrictiveness of ROO often focus on their impact on trade flows. It is clear that more restrictive rules of origin hinder preferential exports, and there is a large empirical literature measuring how large this negative impact is.^5^ Arguably, the more interesting question is how the value-added content in preferential exports of preference beneficiaries is affected by more restrictive rules of origin. Our conceptual framework suggests that the answer to this question is ambiguous. More restrictive ROO unambiguously reduce beneficiaries' preferential exports, but they can simultaneously increase the value-added embedded in preferential exports.

Finally, our results shed some light on the political economy of rules of origin in international trade negotiations. While it is well understood that ROO help protect domestic producers in the preference-granting country (see, for example [6]), it is often ignored that they also help domestic producers of intermediate goods in the beneficiary country. Thus, ROO align the economic interests of domestic producers in the preference donor country with those of the intermediate-good producers in the beneficiary country. Thus, more restrictive rules of origin can expand the set of bilateral trade agreements that are politically feasible.^6^ Interestingly, while beneficiaries of preferential schemes do not always benefit from more lenient rules of origin, foreign providers of intermediate inputs unambiguously benefit. This explains why WTO members that export intermediate inputs to GSP beneficiaries, such as China, often support requests for more lenient ROO in GSP schemes.^7^

Our main challenge consisted of developing a conceptual and empirical framework that would allow us to estimate the beneficiaries' value-added maximizing ROO. The main hurdle is the absence of data on beneficiaries' value-added content in preferential exports. While datasets such as UNCTAD's EORA provide the domestic value-added content of exports at the tariff line level, their estimates are based on input-output tables that, in the case of low-income countries, are estimated using input-output tables for high-income countries. Furthermore, they do not disaggregate between preferential and non-preferential exports. Additionally, ROO regimes often allow for cumulating value-added across different beneficiaries. While EORA allows us to calculate the domestic value-added content of exports, it does not provide the beneficiaries' value-added content of exports cumulated across all beneficiaries. To solve this problem, we developed a framework that estimates the optimal ROO using existing data on preferential exports and ROO. This required us to impose assumptions on the relationship between beneficiaries' value-added content and ROO, discussed in Section 2 and for which we offer some robustness tests. The result is a methodology that allows us to calculate the optimal ROO that maximizes the (unobserved) beneficiaries' value-added content in preferential exports.

We are not the first to look at the impact of ROO on beneficiaries' value-added content in preferential exports [13]. offers seminal theoretical work in this area, modelling the impact on exporter's value-added of domestic value-added content requirements, common in the 1970s and 1980s. It is straightforward to apply his results to the impact of ROO on beneficiaries' value-added content on preferential exports. Using a model with rich micro-foundations [13], shows that the impact is ambiguous; the sign depends on the sign of the elasticity of substitution between domestic factors of production and intermediate imported inputs. If domestic factors of production and imported intermediate inputs are complements (and the share of imported intermediate inputs in total value-added is large) then an increase in the domestic content requirements increases the production costs of the final good disproportionately, leading to a reduction in the total domestic value-added content of exports. Our empirical objective focuses on estimating the optimal ROO (or domestic content requirement in Ref. [13]). We, therefore, do not model the mechanisms in Ref. [13], but the determinants identified in his paper are embedded in our approach and can explain the empirical results in our paper.

[20] examine the rapid rise and fall of Chinese African apparel exports under AGOA (the US GSP scheme for African countries). A significant aspect of AGOA's preferential scheme for apparel was the extremely lenient ROO faced by African exporters located in countries with a GDP per capita below US$1500.^8^ This “loophole” provided an opportunity for Chinese apparel producers, who were constrained in their exports to the United States by the WTO's MFA quotas, to tranship their products through a few African countries, leading to a rapid increase in Lesotho, Madagascar, and Kenya's preferential exports of apparel to the United States. At its peak, over 80% of apparel producers in Kenya's export processing zone were Chinese. Most of these firms were assembly firms. Perhaps more dramatically, when the complete phase-out of MFA quotas arrived in 2005, and Chinese exporters no longer faced quantitative restrictions in the US market, they left the African continent as quickly as they arrived. African exports of apparel to the US under AGOA collapsed [20]. provide systematic evidence that the rise and fall in African apparel exports under AGOA was stronger in tariff lines where Chinese exporters were more quota-constrained in the US market, illustrating the role of footloose Chinese capital in the rapid increase and fall in African exports [20]. indirectly illustrate how very loose ROO can lead to a rapid increase in exports but also very little domestic value-added embedded in those exports. These two concepts are crucial in our conceptual framework and for which our empirical methodology offers a direct test.

The paper closest to ours is [14]. They are the first to show that the relationship between the restrictiveness of ROO and beneficiaries' value-added content is non-monotonic. They call it the ROO Laffer curve because it has the same inverted U-shape as the Laffer curve. It implies that there is an optimal level of ROO that maximizes the beneficiaries' value-added content. Their quantitative model has very rich micro-foundations, allowing for firm heterogeneity across different dimensions and firm location choices within several trade agreement partners. They show that the optimal level depends on the extent of tariff preference granted under preferential market access and the level and variance of cost advantage of foreign suppliers relative to domestic suppliers. They apply the model to assess how the increase in ROO value-added requirements in the automobile sector in the US, Mexico, and Canada Free Trade Agreement (from 62.5% under NAFTA to 75% under USMCA) affects the costs of heterogeneous firms with different costs and sourcing behaviour and ultimately beneficiaries' value-added content. Their paper aims to quantify changes in the cost of compliance at the firm level when ROO change. Our paper builds on [14] ideas. It provides a methodology to calculate optimal ROO that requires data on only ROO and preferential exports, which makes it more feasible to apply to low-income countries where detailed firm-level data is rare.

Finally [19], proposes a property rights framework in which rules of origin help solve the hold-up problem in suppliers' investment decisions to match buyers' needs. In this framework, sufficiently strict ROO enhance welfare, suggesting again that a move towards more lenient ROO is not always in the interest of preferential trade agreements' members. While [19] provides an additional rationale for not moving towards too lenient ROO, this is not a mechanism we consider in our paper. Our setup has no market failures, and the focus is on preferential exporters' value-added.

The remainder of the paper is organized as follows: Section 2 develops a conceptual framework that helps us understand the non-monotonic relationship between beneficiaries' value-added in preferential exports and the restrictiveness of ROO. More importantly, it provides a framework to estimate the optimal ROO without data on value-added content. Section 3 presents the empirical methodology used to estimate the optimal ROO that maximizes value-added in preferential exports. Section 4 discusses data sources and presents descriptive statistics. Section 5 presents our empirical results and Section 6 offers some concluding remarks.

## 2 Conceptual framework

The value-added embedded in beneficiaries' preferential exports is given by:

(1) BVA(r)=α(r)x(r)

where x is the value of preferential exports, BVA is beneficiaries' value-added in preferential exports, α is the share of beneficiaries' value-added in preferential exports, and r is the minimum level of beneficiaries' value-added required by ROO. We are interested in beneficiaries' value-added rather than domestic value-added to capture that preferential regimes such as those granted under the EU'GSP allow for the cumulation of value-added across beneficiaries.^9^

**Fig. 1:**
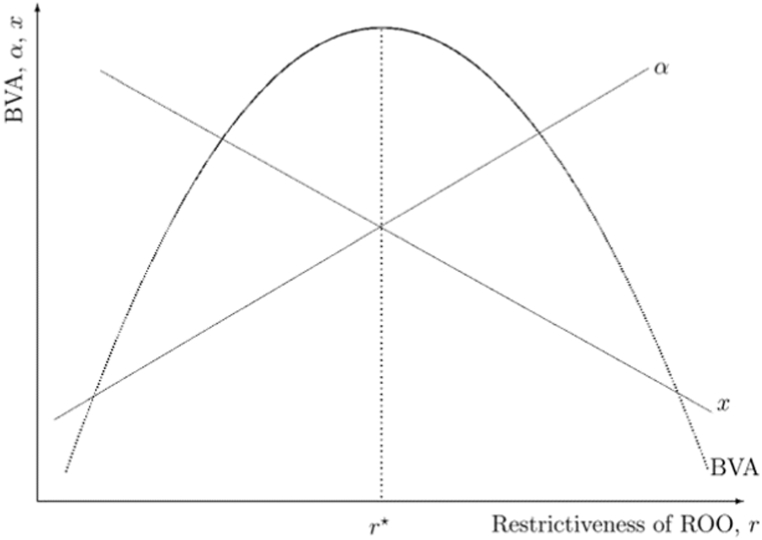
Restrictiveness of ROO and beneficiaries value-added in preferential exports.

Both α and x depend on r. Let us first focus on α. As r becomes more restrictive, α increases. As discussed in Ref. [14], we can decompose this effect into three sub-components. First, there are firms that choose an α that is above the level required by r and remain unconstrained as r becomes more restrictive. Their α is unchanged. Second, there are firms that always comply with the rule of origin, and therefore as r increases α increases. Finally, there are firms which were complying with the low r, but are no longer complying with the higher r. This will also imply that a larger r leads to a higher α due to a composition effect as the firms dropping are those which initially had a lower α. If we combine the three effects, α increases with r. Fig. 1 illustrates the monotonically increasing relationship between α and r.

Preferential exports (x) decline with r. Indeed, as r becomes more restrictive, it increases production cost, leading to a decline in x. This is also illustrated in Fig. 1 with the monotonically decreasing relationship between x and r. These opposing and monotonic relationships between α and x on the one hand, and r on the other hand, lead to a non-monotonic relationship between beneficiaries' value-added content in preferential exports (BVA=αx) and r as shown in Fig. 1. At $r^{*}$, beneficiaries' value-added in preferential exports is maximized.^10^

Taking the derivative of [1] with respect to r, and solving the first order condition for the maximization of BVA, we have that:

(2) $$r^{*}=\underset{r}{\text{argsolve}}1+\frac{\alpha }{\alpha^{\prime }}\frac{x^{\prime }}{x}=0$$

where $\alpha^{\prime }=\partial \alpha /\partial r$ and $x^{\prime }=\partial x/\partial r$.

If we are to apply equation [2] to the data and estimate the BVA-maximizing r, we need to overcome the fact that we do not observe beneficiaries' value-added in preferential exports (BVA) nor its share in preferential exports (α). We only observe preferential exports x. To circumvent this, let us assume that α is a linear function of r.

(3) α=br

where b≥1 when r is expressed in terms of percentage of value-added content, as α cannot be smaller than r, i.e., α≥r. Otherwise, shipments do not qualify for preferential treatment. Below we discuss the implications of relaxing the linearity assumption.

Note that ROO can take other forms, such as a change of chapter or sub-heading of the harmonized system, or the necessity to include certain products, or even be wholly produced. In the conceptual framework, we do not take a stand regarding which form ROO take, as it is conceptually irrelevant to our purposes. It only matters for the interpretation of b, of course. However, in the empirical part, we first present results using ROO that are only defined in terms of value-added content and then check the robustness of our results using data on other forms of ROO.

Replacing equation [3] and its derivative with respect to r into [2], and solving for r, we obtain the BVA-maximizing ROO^11^:

(4) $$r^{*}=-\frac{x}{x^{\prime }}$$

where $x^{\prime }$ stands for ∂x/∂r. x is observable in the data and $x^{\prime }$ can be estimated using data on x and r.

To sum up, the relationship between r and BVA is an inverted-U shape with BVA increasing with r when ROO are lenient, and then decreasing with r as ROO become more restrictive. This suggests that whether more lenient ROO benefit the preference-receiving countries in terms of increasing beneficiaries' value-added content in their preferential exports is an empirical question. It depends on whether the initial restrictiveness of the ROO is set to the left or the right of $r^{*}$.

### 2.1 Relaxing linearity assumption

We relax the assumption that the relationship between α and r is linear to assess the extent to which different functional forms quantitatively matter when calculating the optimal ROO level. In Section 5, we will show that all alternative functional form assumptions yield expressions that are quantitatively very similar to the value obtained using equation [4].

We require alternative functional forms to satisfy five conditions. First, α≥r∀r, meaning that the share of beneficiaries' value-added embedded in preferential exports needs to be larger than required by ROO. Second, 0≤α≤1, to exclude cases that would make no economic sense. Third, $\lim_{r\to 0}\alpha \left(r\right)=0$, recognising that in the absence of any ROO restriction, arbitrage across markets will lead to preferential exports being completely sourced from lower-cost producers in the rest of the world. Fourth, the parameters of the function need to cancel out in the first order condition, as we are not able to identify the parameters of the α function without data on α. Finally, the second-order condition for a maximum needs to be satisfied.

We consider three alternative relationships between α and r that satisfy these five conditions: a logarithmic relationship, a square root relationship, and a geometric relationship. Let us start with a logarithmic function:

(5) α=bln(1+r)

Taking the derivative of [5], we have $\alpha^{\prime }=b/\left(1+r\right)$. Rearranging after substituting α and $\alpha^{\prime }$ into the right-hand-side of the first equality in equation [2], we obtain:

(6) $$\left(1+r^{*}\right)\ln\left(1+r^{*}\right)=-\frac{x}{x^{\prime }}$$

A first order Taylor series approximation to the left-hand-side of [6] for values of $r^{*}$ close to 0 yields:

(7) $$r^{*}\approx -\frac{x}{x^{\prime }}$$

Thus, comparing equations [4,7], we conclude that a linear approximation of the optimal ROO under the logarithmic function assumption yields the same optimal ROO as when assuming a linear relationship between α and r, at least for small values of r.

But r sometimes takes values much larger than 0, and therefore the first-order Taylor series approximation may be misleading. An exact solution for $r^{*}$ in equation [6] requires using the Lambert W function and is given by^12^:

(8) $$r^{*}=-\frac{\frac{x}{x^{\prime }}+\text{LambertW}\left(-\frac{x}{x^{\prime }}\right)}{\text{LambertW}\left(-\frac{x}{x^{\prime }}\right)}$$

The second alternative we consider is that the relationship between α and r is given by the following square root function:

(9) $$\alpha =b\sqrt{r}$$

Taking the derivative of [9], we have $\alpha^{\prime }=b/\left(2\sqrt{r}\right)$. Rearranging after substituting α and $\alpha^{\prime }$ into the right-hand-side of the first equality in equation [2], we obtain:

(10) $$r^{*}=\sqrt{-\frac{x}{x^{\prime }}}=0$$

Equation [10] is the BVA-maximizing rule of origin when the relationship between α and r is given by a square root function.

The final alternative functional form we consider assumes that the relationship between α and r follows a geometric series:

(11) $$\alpha =b\left(r+r^{2}-r^{3}\right)$$

Taking the derivative of [11], we have $\alpha^{\prime }=1+2r-3r^{2}$. Rearranging after substituting α and $\alpha^{\prime }$ into the right-hand-side of the first equality in equation [2], we obtain:

(12) $$\frac{r+r^{2}-r^{3}}{1+2r-3r^{2}}=-\frac{x}{x^{\prime }}$$

Equation [12] has no closed-form solution for r, but we will solve it numerically with estimates for $-x/x^{\prime }$, i.e., the inverse of the semi-elasticity of x with respect to r. In Section 3, we discuss our strategy for obtaining estimates for each case.

**Fig. 2:**
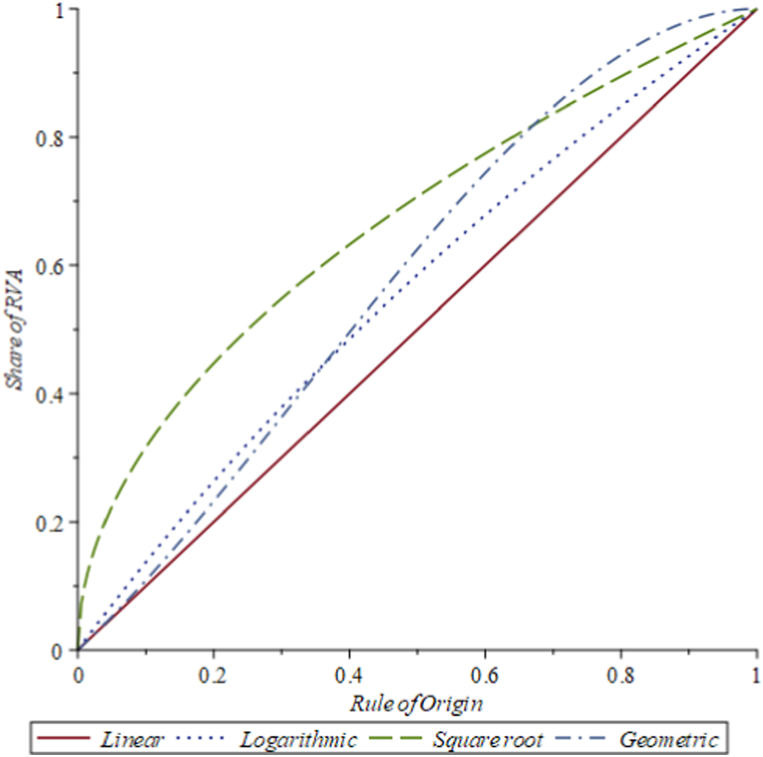
Relationship between share of Beneficiaries Value-Added (α) and ROO (r). **Note**: The linear relationship assumes α=br, the logarithmic relationship assumes α=bln(1+r), the square root relationship assumes $\alpha =b\sqrt{r}$, and the geometric relationship assumes $\alpha =b\left(r+r^{2}-r^{3}\right)$. In each case the parameter b is chosen so that 1≥α≥r≥0.

To visualize the implications of the four different functional form assumptions for the relationship between α and r, we plot them in Fig. 2, choosing b in each case so that the five conditions imposed above are satisfied. In the case of the linear relationship, this implies that the rule of origin is always binding, whereas in the three alternative assumptions (logarithmic, square root, and geometric functions) α can be larger than r. It is also clear that the four functional forms allow for substantial differences in the relationship between α and r. If quantitatively, the optimal ROO are quite similar, functional form uncertainty should not be a concern. In the results section, we will discuss the sensitivity of predictions to variations in these assumptions in the spirit of the interval predictions of policy outcomes in [18].

### 2.2 Impact on foreign value-added

The rest of the world exporters of intermediate inputs to preference beneficiary countries benefit unambiguously from more lenient ROO. To illustrate this, decompose preferential exports into beneficiaries and foreign value-added (rest of the world) content:

(13) x=BVA+FVA

Replace equation [1] into equation [13] and solve for FVA to obtain:

(14) FVA=(1−α)x

Take the derivative of both sides of equation [14] with respect to r to obtain:

(15) $${\text{FVA}}^{\prime }=\left(1-\alpha \right)x\prime -\alpha^{\prime }x\leq 0$$

Indeed ${\text{FVA}}^{\prime }\leq 0$ in equation [15] as x′≤0 and $\alpha^{\prime }\geq 0$. Intuitively, as ROO become more lenient, this boosts FVA through two channels. First, a more lenient ROO implies larger preferential exports, which increases the demand for foreign intermediary inputs. Second, more lenient ROO allow for a larger share of foreign inputs embedded in preferential exports, again leading to an increase in the demand for foreign intermediary inputs. Therefore, the impact on rest of the world exporters of intermediary inputs to beneficiary countries is unambiguously positive. Whether foreign value-added owners benefit from more lenient ROO is not an empirical question, in contrast to what we discussed above for preference beneficiaries.

## 3 Empirical framework

To estimate the relationship between BVA and r, we would ideally use data on value-added embedded in preferential exports. Unfortunately, this data is not available for GSP beneficiaries. However, using the structure and assumptions of the previous section, we can circumvent this problem. Under all the alternative assumptions assumed in the previous section, the optimal ROO is a function of the semi-elasticity of preferential exports with respect to r, i.e., $x^{\prime }/x$. Thus, we only need data on preferential exports and r to estimate the optimal ROO. Both of these are available.

To directly estimate the semi-elasticity of preferential exports with respect to r, we use the gravity model of trade. The model explains the variation in bilateral trade using supply (exporting country) and demand (importing country) factors as well as bilateral trade costs. We use a Poisson estimator to address the presence of zero trade flows, which, in the presence of heteroskedasticity, can lead to biased estimates when variables are log-linearized [21, 24]. We focus on the European Union's GSP schemes and treat the European Union as one importer, which implies that there is no variation in the data across importers. More formally:

(16) $$x_{xpt}=e^{\beta_{xt}+\beta_{pt}+\beta_{xp}+\beta^{T}\;\ln\frac{1+T_{pt}}{1+t_{xpt}}+\beta^{\alpha }\alpha_{xpt}}+\varepsilon_{xpt}$$

where $x_{xpt}$ are preferential exports from country x to the European Union of product p at time t, and β s are parameters to be estimated by Poisson quasi-pseudo maximum likelihood; $\beta_{xt}$ are exporter × time fixed effects, $\beta_{pt}$ are product × time fixed effects, and $\beta_{xp}$ are exporter × product fixed effects to address omitted variable concerns that could explain both the restrictiveness of ROO and bilateral exports; $\varepsilon_{xpt}$ is an i. i.d error term.

Our model includes two bilateral trade cost variables: the preferential tariff margin and the share of beneficiaries' value-added content in preferential exports. The preferential tariff margin is captured by the ratio of MFN to preferential tariffs ($\left(1+T_{pt})/(1+t_{xpt}\right)$).^13^

Our variable of interest is the share of value-added in preferential exports ($\alpha_{xpt}$), which we do not observe, but is a function of $r_{xpt}$. Thus we replace $\alpha_{xpt}$ with each of the functional forms in equations [3,5,9,11] and introduce them into equation [16] to obtain:

(17) $$x_{xpt}=e^{\gamma_{xt}+\gamma_{pt}+\gamma_{xp}+\gamma^{T}\;\ln\frac{1+T_{pt}}{1+t_{xpt}}+\gamma^{r}r_{xpt}}+\mu_{xpt}$$

(18) $$x_{xpt}=e^{\delta_{xt}+\delta_{pt}+\delta_{xp}+\delta^{T}\;\ln\frac{1+T_{pt}}{1+t_{xpt}}+\delta^{r}\;\ln\left(1+r_{xpt}\right)}+u_{xpt}$$

(19) $$x_{xpt}=e^{\psi_{xt}+\psi_{pt}+\psi_{xp}+\beta^{T}\;\ln\frac{1+T_{pt}}{1+t_{xpt}}+\psi^{r}\sqrt{r_{xpt}}}+\omega_{xpt}$$

(20) $$x_{xpt}=e^{\zeta_{xt}+\zeta_{pt}+\zeta_{xp}+\zeta^{T}\;\ln\frac{1+T_{pt}}{1+t_{xpt}}+\zeta^{r}\left(r_{xpt}+r_{xpt}^{2}\right)-r_{xpt}^{3}}+\xi_{xpt}$$

where $\gamma^{r}$, $\delta^{r}$, $\psi^{r}$ and $\zeta^{r}$ are now the parameters of interest (that incorporate the b parameter in the different functions of α).

The semi-elasticity of preferential exports with respect to r are then given by:

(21) $$\frac{x^{'}}{x}=\gamma^{r}\;\text{if}\;\alpha =\text{br}$$

(22) $$\frac{x^{'}}{x}=\frac{\delta^{r}}{1+r}\;\text{if}\;\alpha \;=\;\text{bln}\left(1+r\right)$$

(23) $$\frac{x^{'}}{x}=\psi^{r}e^{r-1}\;\text{if}\;\alpha =be^{r-1}$$

(24) $$\frac{x^{'}}{x}=\zeta^{r}\left(1+2r-3r^{2}\right)\;\text{if}\;\alpha =b\left(r+r^{2}-r^{3}\right)$$

Substituting equation [21] into equation [4], equation [22] into equation [8], equation [23] into equation [10], and equation [24] into equation [11] we obtain the optimal ROO, $r^{*}$, under the four different functional forms:

(25) $$r^{*}=-\frac{1}{\gamma^{r}}\;\text{if}\;\alpha =\text{br}$$

(26) $$r^{*}=e^{-\frac{1}{\delta^{r}}}-1\;\text{if}\;\alpha =\text{bln}\left(1+r\right)$$

(27) $$r^{*}=\sqrt{\left(-\frac{1}{\psi^{r}}\right)}\;\text{if}\;\alpha =b\sqrt{r}$$

(28) $$r^{*}=\underset{r}{\text{argsolve }}r+r^{2}-r^{3}=-\frac{1}{\zeta^{r}}\;\text{if}\;\alpha =b\left(r+r^{2}-r^{3}\right)$$

Note that in the case of a geometric series, there is no closed-form solution for $r^{*}$, but we will solve equation (28) numerically.

The standard error of $r^{*}$ in equations (25), (26), (27), (28) is calculated using a first-order Taylor series approximation of $r^{*}$ around its mean..^14^

(29) $$se\left(r^{*}\right)=se\left(\gamma^{r}\right)\frac{1}{{\left(\hat{\gamma^{r}}\right)}^{2}}$$

(30) $$se\left(r^{*}\right)=se\left(\delta^{r}\right)\frac{e^{-\frac{1}{\hat{\delta^{r}}}}}{{\left(\hat{\delta^{r}}\right)}^{2}}$$

(31) $$se\left(r^{*}\right)=se\left(\psi^{r}\right)\left(\frac{1}{2{\left(-\hat{\psi^{r}}\right)}^{\frac{3}{2}}}\right)$$

(32) $$se\left(r^{*}\right)=se\left(\zeta^{r}\right)\left(\frac{1+2r^{*}-3{r^{*}}^{2}}{{\hat{\zeta^{r}}}^{2}}\right)$$

where se stands for standard error.

Equations [[17], [18], [19], [20]] are estimated using preferential exports on the left-hand-side and not all bilateral exports, contrary to most of the existing empirical literature on ROO.^15^ This is important because otherwise, we would capture the impact of ROO on both preferential and non-preferential exports. These two effects work in different directions, and we are interested in the first effect, as those are the flows that are relevant and targeted by preferential schemes. Indeed, the increase in non-preferential flows associated with an increase in the restrictiveness of ROO would have also existed in the absence of a preferential scheme and therefore the additional value-added cannot be attributed to changes in preferential market access. In other words, if the counterfactual is exports that would have existed in the absence of a preferential regime, the change in beneficiaries' value-added associated with ROO that leads to changes in non-preferential exports should not be taken into account.

If we were to work with total exports, the optimal ROO would be higher than the ones we calculate using preferential exports, as long as an increase in the restrictiveness of rules of origin increases non-preferential exports. To see this, denote $x_{T}=x+x_{np}$, where $x_{T}$ are total exports and $x_{np}$ are non-preferential exports, then $x_{np}^{\prime }\geq 0$, where $x_{np}^{\prime }$ is the derivative of non-preferential exports with respect to the ROO r. Therefore, when adding $\alpha_{np}^{\prime }x_{np}+\alpha_{np}x_{np}^{\prime }$ to the right-hand side of the first equality in equation [2], we are adding two positive terms, and therefore when solving for the optimal r, we should obtain a higher $r^{*}$ than the one obtained with [3] when using preferential exports only.

To verify that $x_{np}^{\prime }\geq 0$, in the results section we also report estimates for non-preferential exports using the same models as in equations [[17], [18], [19], [20]], but with non-preferential exports on the left-hand-side instead of preferential exports. We expect the parameters in front of r to be positive when running equations [[17], [18], [19], [20]] on non-preferential exports, as stronger requirements on beneficiaries' value-added content reduce preferential exports, as they become more costly, increasing the incentives to export non-preferentially even though it involves paying MFN tariffs in the importing country.

In equations [[17], [18], [19], [20]], the parameters in front of r are identified using the variation across years within exporter × product, across exporters within product × year, and across products within exporter × year. Thus, to identify the semi-elasticity of preferential exports with respect to rules of origin, we need to have variation in ROO along these three dimensions. This implies that we need to have changes in ROO across time (within products × exporter). This is the reason we choose the EU's GSP schemes to estimate equations [[17], [18], [19], [20]]. The EU is a major donor in terms of preferential market access and has implemented significant ROO reforms in recent years.^16^ In the absence of time variation, ROO will be perfectly collinear with the exporter × product fixed effects and we would not be able to identify our parameters of interest. We also need to have variation in ROO across exporters (within product × year), and this implies that we need to consider more than one preferential scheme. The EU's GSP preferences are again ideal because it has three sub-regimes: the standard GSP, the GSP+, and the Everything But Arms (EBA) regime granted to LDCs.^17^ If we were to estimate equations [[17], [18], [19], [20]] on data for EBA beneficiaries only, we would not be able to identify our parameters of interest. All exporters would face the same ROO, which will then be perfectly collinear with the product × year fixed effects. Finally, we need variation across products.^18^ Otherwise, the ROO will be perfectly collinear with the exporter × year fixed effect. The EU's GSP has sufficient variation across tariff lines, unlike other countries' GSP regimes such as Australia, New Zealand, or Taiwan, which all have a 50% value-added requirement across all products.

## 4 Data

Our dataset covers the period from 2008 to 2019 and is disaggregated at the six-digit level of the Harmonized System (HS). Below, we provide data sources and descriptive statistics for ROO, tariffs, trade, and preference utilization data.

### 4.1 ROO

Data for ROO is available for all preferential schemes through the International Trade Centre (ITC)-World Customs Organization (WCO)-WTO's ROO Facilitator database and is coded based on member states' notifications.^19^ The value-added criteria is one of the most popular criteria used to determine origin, and is expressed as the minimum share of beneficiaries' value-added that needs to be embedded in preferential exports to obtain origin and benefit from preferential access.^20^ It is the criteria on which we focus our discussion in the results section.

However, other substantial transformation criteria are also used to determine origin. Change of tariff headings and technical regulations are equally popular criteria in the EU's GSP regime. Our conceptual framework does not rely on the use of a value-added criterion, and therefore we will apply our framework to a measure of ROO restrictiveness that incorporates all possible forms of ROO. This restrictiveness index (R-index) ranks the stringency of all origin criteria between 0 and 1 for each product/year/regime combination. The R-index we used is documented in Ref. [17], which is based on the pioneering work by Refs. [8,12,15].

Columns 1 and 2 in Table 1 provide summary statistics for the value-added criteria of ROO and the R-index faced by EU's GSP beneficiaries (top panel) and LDC beneficiaries only (bottom panel). For the period under examination, the average value-added criteria in ROO faced by GSP exporters to the EU is 46%, whereas LDC face a lower threshold of 39%. The median value-added criteria faced by GSP beneficiaries is significantly higher than LDC beneficiaries at 50 and 30% respectively. However, when looking at the restrictiveness of the R-index that encompasses all ROO criteria, the restrictiveness of ROO does not seem to be very different between GSP and LDC beneficiaries, indicating that the more flexible ROO for LDC are only observed for the value-added criteria.

**Table 1:** Summary statistics for European Union's GSP and LDC preferential regimes.^a^.

	Value-added	R-index	Preference	Total	Preferential	Non-Pref.
	ROO		Margin^b^	Exports^c^	Exports^d^	Exports^e^
	Top panel					
	All GSP beneficiaries					
Mean	0.46	0.25	1.04	0.27	0.16	0.11
Standard deviation	0.13	0.20	0.02	8.27	5.22	3.95
Median	0.50	0.20	1.04	0.00	0.00	0.00
Minimum	0.30	0.00	1.00	0.00	0.00	0.00
Maximum	0.60	0.95	1.14	1810	983	826
	Bottom panel					
	LDC beneficiaries only					
Mean	0.39	0.25	1.05	0.82	0.69	0.14
Standard deviation	0.13	0.24	0.02	14.02	11.63	2.58
Median	0.30	0.20	1.05	0.00	0.00	0.00
Minimum	0.30	0.00	1.01	0.00	0.00	0.00
Maximum	0.60	0.95	1.15	366	296	81.2

a **Note:** In the top panel we report these statistics in a sample with all GSP beneficiaries, and in the bottom panel for only LDC beneficiaries. The data spans from 2008 to 2019.

b It is defined as the (1 + T)/(1 + t) which takes the value 1 in the absence of margin (t = T) and a value larger than 1 presence of a preference margin (t < T).

c Total exports are measured in millions of US dollars.

d Preferential exports are measured in millions of US dollars.

e Non-preferential exports are measured in millions of US dollars.

### 4.2 Tariff data

Data on EU's MFN and GSP preferential tariffs under different regimes (LDC, GSP+, and standard GSP in the case of the EU) was obtained from the International Trade Centre's Market Access Map. The tariff data is collected directly from national authorities by the ITC every year and was downloaded from https://www.macmap.org/en/download. In the case of specific duties (e.g. 5 USD per 1 L) or tariff rate quotas, the ITC performs a conversion into an ad-valorem equivalent using a methodology described in World Tariff Profiles (2008). The preference margin is constructed as the ratio of 1 plus the MFN tariff divided by 1 + the preferential tariff. In the absence of preferences, the preference margin equals 1 and is higher otherwise.

Column 3 in Table 1 provides summary statistics for the log of the preference margin granted to all of the EU's GSP beneficiaries (top panel) and LDC beneficiaries only (bottom panel). The average and median preference margin during the period under examination is 4% for GSP beneficiaries and 5% for LDC beneficiaries. It varies between 0 and 15%.

### 4.3 Exports

Data on preferential and non-preferential exports is available through the WTO's Integrated Data Base's Tariff Analysis Online facility, which is accessible at https://tao.wto.org. The data is provided by WTO member states every year and it provides a breakdown by preferential regime. In the case of the EU, the preference utilization data is provided by EUROSTAT. Columns 4 to 6 in Table 1 provide summary statistics for total exports, preferential exports, and non-preferential exports for all EU's GSP beneficiaries (top panel) and LDC beneficiaries only (bottom panel).

GSP beneficiaries' average preferential exports are USD 0.16 million. Non-preferential exports are, on average, USD 0.11 million for total exports of USD 0.27 million. Thus, on average during the period under examination, 59% of GSP beneficiaries' exports to the EU enter preferentially. In the case of LDC beneficiaries, 84% of exports enter the EU preferentially, signaling a more generous preferential regime.

## 5 Results

We report the results of the estimation of equations [[17], [18], [19], [20]] for both preferential and non-preferential exports of GSP beneficiaries to the EU. Table 2, Table 3 report results using data at the 6-digit and 4-digit levels of the HS, respectively. We report results at the 4-digit level because this is the level of aggregation at which ROO are set in the EU. In both tables, the first two columns present results assuming that there is a linear relationship between α and r. Columns [3,4] present results assuming a logarithmic relationship, columns [5,6] assume a square root relationship, and the last two columns a geometric relationship. In Table 2, Table 3, ROO are captured using value-added requirements. We later test the robustness of results using the comprehensive index of ROO restrictiveness, the R-index.

**Table 2:** Value-added ROO and GSP exports to the EU.^a^.

	Linear		Logarithmic		Square root		Geometric	
	α=br		α=bln(1+r)		$\alpha =b\sqrt{r}$		$\alpha =b\left(r+r^{2}-r^{3}\right)$	
	Pref. exp.	Non-pref.	Pref. exp.	Non-pref.	Pref. exp.	Non-pref.	Pref. exp.	Non-pref.
		exports		exports		exports		exports
	[1]	[2]	[3]	[4]	[5]	[6]	[7]	[8]
Preference margin	−2.720	−0.943	−2.720	−0.943	−2.720	−0.943	−2.720	−0.943
	(2.082)	(3.307)	(2.082)	(3.307)	(2.082)	(3.307)	(2.082)	(3.307)
ROO	−2.679∗	3.813∗	−3.745∗	5.330∗	−3.362∗	4.785∗	−2.046∗	2.910∗
	(0.816)	(0.928)	(1.140)	(1.297)	(1.024)	(1.165)	(0.623)	(0.709)
Observations	22632	116193	22632	116193	22632	116193	22632	116193
Pseudo R^2^	0.984	0.979	0.984	0.979	0.984	0.979	0.984	0.979

a **Note:** All regressions use a pseudo Poisson maximum likelihood estimator and contain exporter × year, product × exporter, and product × year fixed effects. Statistical significance is indicated with superscript * for p-value <0.001, ⊛ for p-value <0.01, and † for p-value <0.05. In all regressions, standard errors are clustered at the 4-digit of the HS times year level to account for the fact that this is the level at which ROO are set in the EU. The first two columns provide results assuming a linear relationship between α and r, the third and fourth columns assume a logarithmic relationship, the fifth and sixth columns a square root relationship, and the last two columns a geometric relationship.

**Table 3:** Value-added ROO and GSP exports to the EU at the 4- digit level of the HS.^a^.

	Linear		Logarithmic		Square root		Geometric	
	α=br		α=bln(1+r)		$\alpha =b\sqrt{r}$		$\alpha =b\left(r+r^{2}-r^{3}\right)$	
	Pref. exp.	Non-pref.	Pref. exp.	Non-pref.	Pref. exp.	Non-pref.	Pref. exp.	Non-pref.
		exports		exports		exports		exports
	[1]	[2]	[3]	[4]	[5]	[6]	[7]	[8]
Preference margin	6.088∗	−8.296†	6.088∗	−8.296^⊛^	6.088∗	−8.296^⊛^	6.088∗	−8.296^⊛^
	(1.441)	(3.591)	(1.441)	(3.186)	(1.441)	(3.186)	(1.441)	(3.186)
ROO	−2.467∗	5.257∗	−3.448∗	7.347∗	−3.096∗	6.596∗	−1.833∗	4.013∗
	(0.761)	(1.410)	(1.140)	(1.455)	(0.955)	(1.301)	(0.581)	(0.795)
Observations	10361	47841	10361	47841	10361	47841	10361	47841
Pseudo R^2^	0.991	0.982	0.991	0.982	0.991	0.982	0.991	0.982

a **Note:** All regressions use a pseudo Poisson maximum likelihood estimator and contain exporter × year, product × exporter, and product × year fixed effects. Statistical significance is indicated with superscript * for p-value <0.001, ⊛ for p-value <0.01, and † for p-value <0.05. In all regressions, standard errors are clustered at the 4-digit of the HS times year level to account for the fact that this is the level at which ROO are set in the EU. The first two columns provide results assuming a linear relationship between α and r, the third and fourth columns assume a logarithmic relationship, the fifth and sixth columns a square root relationship, and the last two columns a geometric relationship.

The coefficients on ROO in Table 2, Table 3 always have the expected sign and are statistically significant at the 0.1% level. As expected, more restrictive value-added requirements reduce preferential exports and increase non-preferential exports. Note also that when comparing ROO coefficients for the same specifications across Table 2, Table 3, they tend to be very similar. In particular, the coefficients on ROO in the regressions of preferential exports (odd number columns), which are the relevant coefficients for our estimates of optimal ROO, are all within one standard deviation of each other when comparing estimates at the 6 and 4-digit levels in Table 2, Table 3, respectively. In the case of preferential tariff margins, only the estimates at the 4-digit of the HS are statistically significant, which can be explained by the lack of variation in the data at the 6-digit of the HS. Indeed, most preferential tariffs are equal to 0 across all goods and exporters, which implies that the preference margin is just given by the MFN tariff, which is in turn perfectly collinear with product × year fixed effects. At the 4-digit, there is more variation by construction as the MFN tariff is computed using import-weights at the 6-digit level. For this reason, the results on the preferential margins should be interpreted with care. In any case, results in Table 3 show that an increase in preferential tariff margins increases preferential exports and decreases non-preferential exports, as expected.

Using the estimated coefficients for ROO in the regressions of preferential exports (odd-number columns) in Table 2, Table 3, we can compute the optimal ROO, $r^{*}$. The optimal ROO depends on the assumptions discussed in Section 2 regarding the relationship between the share of beneficiaries' value-added in preferential exports (α) and the ROO r. The optimal values are given by equations (25), (26), (27), (28).

Table 4 provides the values of the optimal ROO under the four different function form assumptions for estimates at the 6 and 4-digit level of the HS. Using the baseline assumption of a linear relationship between α and r, we obtain $r^{*}=0.37$ when using data at the 6-digit of the HS, and 0.41 when using data at the 4-digit of the HS (first column estimates in Table 2, Table 3). This implies that for any initial ROO larger than these optimal values, a more lenient ROO will lead to an increase in beneficiaries' value-added. However, for initial values that are smaller than these optimal values, a more lenient ROO will lead to a decrease in beneficiaries' value-added.

**Table 4:** Optimal ROO maximizing beneficiaries' value-added ($r^{*}$)^a^.

	HS 6-digit	HS 4-digit
Assumption α = *f*(*r*)		
Linear function^b^	0.37	0.41
	(0.11)	(0.13)
Logarithmic function^c^	0.31	0.34
	(0.11)	(0.12)
Square root function^d^	0.55	0.57
	(0.08)	(0.09)
Geometric function^e^	0.40	0.43
	(0.20)	(0.21)

a The first column provides the optimal ROO using the estimates for the ROO variable at the 6-digit of the HS in Table 2. The second column provides the optimal ROO using the estimates at the 4-digit of the HS in Table 3. Each row uses a different assumption regarding the relationship between α and r corresponding to the columns in Table 2, Table 3 The first row uses a linear function, the second uses a logarithmic function, the third has a square root function, and the fourth is a geometric function. Numbers in parentheses are standard errors.

b See equation (25) for the optimal ROO and equation (29) for the standard error.

c See equation (26) for the optimal ROO and equation (30) for the standard error.

d See equation (27) for the optimal ROO and equation (31) for the standard error.

e See equation (28) for the optimal ROO that we solved numerically, and equation (32) for the standard error.

Different functional forms lead to different optimal values, as can be seen from Table 3, but the differences are small. The optimal ROO varies from 0.31 to 0.55 when estimated at the 6-digit of the HS, and between 0.34 and 0.57 when estimated at the 4-digit of the HS. The mean estimate at the 6-digit level is 0.41 with a standard deviation of 0.10. At the 4-digit level, the mean estimate is 0.44 with a standard deviation of 0.10. The relatively modest standard deviations across estimates suggest that functional form uncertainty should not be an important concern.

The standard errors of the optimal ROO reported in Table 4 are estimated using equations (29), (30), (31), (32). They are quite precisely estimated in all cases, partly because of the precision with which $\gamma^{r}$, $\delta^{r}$, $\psi^{r}$, and $\zeta^{r}$ are estimated in Table 2, Table 3 Importantly, all optimal values are not statistically different from each other. Measurement uncertainty should not be an important concern either.

Note also that all optimal values are statistically different from 0 to 1, consistent with an interior solution. As discussed in the appendix, the second-order conditions for a maximum are always satisfied at the optimum under the four different functional form assumptions.

The average value of $r^{*}$ across the four specifications is 0.41 when estimated using data at the 6-digit level of the HS, and 0.44 when using data at the 4-digit level. These average values are not very different from the ones obtained in the baseline scenario where we assumed a linear relationship between α and r (0.37 and 0.41). If we compare these values to the average value-added ROO requirement across the EU's GSP regimes of 0.46 (as reported in the first column of Table 1), we are tempted to conclude that value-added requirements in EU's GSP preferential regimes are set not too far away from optimal levels. This implies that there is not much room for further liberalizing some of these requirements in order to increase beneficiaries' value-added embedded in preferential exports. This is also true if we compare the optimal values to the average value-added requirement faced by LDCs in the EU, which, as reported in Tables 1 and is equal to 0.39. These two values (0.46 for GSP and 0.39 for LDC) are within the 95% confidence intervals of all estimated $r^{*}$ reported in Table 4.

We also report results of the estimation of [[17], [18], [19], [20]] but using R-index instead of the value-added criteria as a measure of ROO's restrictiveness. As reported in Table 5, all the estimated coefficients have the expected sign and are statistically significant, although the coefficients on ROO restrictiveness are not as precisely estimated as in Table 2, Table 3

**Table 5:** ROO R-index and GSP exports to the EU.^a^.

	Linear	Logarithmic	Square root	Geometric
	α=br	α=bln(1+r)	$\alpha =b\sqrt{r}$	$\alpha =b\left(r+r^{2}-r^{3}\right)$
	Pref. exp.	Pref. exp.	Pref. exp.	Pref. exp.
	[1]	[2]	[3]	[4]
Preference margin	4.785∗	4.785∗	4.785∗	4.785∗
	(0.676)	(0.676)	(0.676)	(0.676)
ROO	−1.253†	−1.459†	−2.300†	−1.012†
	(0.736)	(0.877)	(1.333)	(0.584)
Observations	237437	237437	237437	237437
Pseudo R^2^	0.983	0.983	0.983	0.983
Optimal ROO^b^ –	0.798	0.985	0.659	0.921
	(0.486)	(0.818)	(0.191)	(0.170)
Average R-index	0.214	0.214	0.214	0.214

a **Note:** All regressions use a pseudo Poisson maximum likelihood estimator and contain exporter × year, product × exporter, and product × year fixed effects. Statistical significance is indicated with superscript * for p-value <0.001, ⊛ for p-value <0.01, and † for p-value <0.05. In all regressions, standard errors are clustered at the 4-digit of the HS times year level to account for the fact that this is the level at which ROO are set in the EU. The first column provides results assuming a linear relationship between α and r, the second column assumes a logarithmic relationship, the third column a square root relationship, and the last column a geometric relationship.

b See equations (25), (26), (27), (28) for the optimal ROO under different functional form assumptions, and (29)–(30) for its standard error.

Using the estimated coefficients reported in Table 5, we can again compute the optimal ROO, not in terms of value-added, but in terms of R-index. Again, note that there is nothing in Section 2 that requires ROO to be expressed in terms of value-added content, so the results in that section extrapolate also to other measures of ROO such as those captured by R-index. The optimal values of $r^{*}$ and their standard errors are provided at the bottom of Table 5, as well as the average R-index in our sample. The optimal values are much larger than the average R-index in the sample, suggesting that moving towards more lenient rules of origin is likely to reduce beneficiaries' value-added in preferential exports. Thus, the case for not moving towards more lenient rules of origin is strengthened when moving towards other ROO criteria, such as change of tariff classification or technical requirements.

## 6 Concluding remarks

Over the last two decades, the LDC group in the WTO has consistently focused its negotiating capital in demands for relaxation of the restrictiveness of Rules of Origin (ROO) granting unilateral preferential access to LDCs. Building on [14], this paper argues that whether beneficiaries benefit or not from more lenient ROO when benefits are measured in terms of beneficiaries' value-added embedded in preferential exports, depends critically on whether the initial ROO is higher or lower than the value-added maximizing ROO.

To assess whether calls for more lenient ROO on preferential regimes for low-income countries would increase beneficiaries' value-added in preferential exports, we estimate the optimal ROO that maximizes beneficiaries' value-added and compare it to the existing restrictiveness in ROO. The average optimal ROO in the EU's GSP regime across different functional forms is estimated at 41%. In 2019 the average ROO value-added requirement in our sample of GSP exporters to the EU is 34%. This is below all estimates of the optimal ROO using different functional form assumptions and levels of aggregation, except for the logarithmic function when estimated at the six-digit level of the HS for which we obtain an optimal value of 31%. Reducing the restrictiveness of ROO to the 15–25% value-added requirement as requested by the LDC group in the WTO is likely to decrease beneficiaries' value-added embedded in preferential exports.

More lenient ROO in the EU GSP scheme is unlikely to benefit those it intends to help. However, as shown in Section 2.2, the rest of the world suppliers of intermediate goods to GSP beneficiaries will unambiguously benefit. This is because more lenient rules of origin not only allow for sourcing larger amounts of inputs from the rest of the world per unit of exports but also reduce production costs in the exporting country, which increases preferential exports and, therefore, demand for inputs provided by rest of the world suppliers.

These results imply that calls for more lenient ROO in the EU GSP scheme should be carefully assessed if the objective is to help beneficiaries. A reduction in the restrictiveness of ROO will definitely help the rest of the world suppliers of intermediate inputs to GSP beneficiaries, but it has the potential to hurt beneficiaries in terms of their value-added content, which can ultimately hurt their growth potential.

Finally, a word of caution: our results are obtained in a sample of unilateral preferential schemes granted by the EU to low-income beneficiaries. It may be tempting to extrapolate our results to bilateral preferential schemes among high-income or low-income countries or even between high and low-income countries. Many institutional and economic fundamental differences between these countries and preferential schemes must be addressed thoroughly before extrapolating the results. More generally, the optimal ROO is likely to vary by industry and depend on beneficiary characteristics and their capacity to add value-added to preferential exports.

## Data availability statement

Data will be made available on request.

## Additional information

No additional information is available for this paper.

## CRediT authorship contribution statement

**Dzmitry Kniahin:** Writing – original draft, Formal analysis, Data curation. **Marcelo Olarreaga:** Writing – review & editing, Writing – original draft, Methodology, Formal analysis, Conceptualization.

## Declaration of competing interest

The authors declare that they have no known competing financial interests or personal relationships that could have appeared to influence the work reported in this paper.

## Appendix Second order conditions

To ensure that the optimal ROO in equations (25), (26), (27), (28) are maxima and not minima, we verify the second-order conditions:

(33) $$\frac{\partial^{2}\text{BVA}}{\partial r^{2}}=x\alpha^{\prime }\prime +2\alpha^{\prime }x^{\prime }+{\alpha x}^{\prime \prime }$$

where $x^{\prime }\leq 0$ as discussed in the text and $\alpha^{\prime \prime }$ and $x^{\prime \prime }$ are the second derivative of α and x with respect to r. So we have that the right-hand-side of equation (33) is negative as long as x and α are not too convex in r.

We numerically verify these conditions at the optimum (i.e, for $r^{*}$) and for the estimated values of the semi-elasticities of preferential exports with respect to α, and found that for the four functional forms the second-order is negative, ensuring that the optimum is a maximum.

## Footnotes

1 The World Trade Organization's (WTO) Enabling Clause of 1979 provides the legal basis for this Most Favoured Nation exemption to the General Agreement on Tariffs and Trade (GATT).

2 The literature focusing on the impact of GSP on beneficiaries' exports suggests a positive and large impact (see [10]).

3 [9] challenge the need for ROO to determine origin arguing that the benefits from current tariff preferences are generally lower than transport costs, making trade deflection economically uninteresting.

4 When Sam Lowe was asked on Twitter to explain what topic he could not stop talking about that would lead kidnappers to prefer to let him go rather than put up with him, he answered: rules of origin (https://twitter.com/SamuelMarcLowe/status/1504588138429231106). In the paragraphs below, we take on the difficult task of convincing the reader that the kidnappers would have made a mistake.

5 See for example [7].

6 Note that this does not apply to unilateral schemes such as the GSP because the preference donor country unilaterally determines them.

7 See [20] for an example of how Chinese providers of intermediate inputs benefited from AGOA's flexible rules of origin.

8 The only requirement was that the products were assembled in the African country.

9 The EU's GSP regime allows for bilateral cumulation of European value-added in beneficiaries' preferential exports, as well as regional cumulation for twelve Asian beneficiaries divided into two regional groups. Our analysis focuses on the maximization of the cumulative value-added as allowed by EU's GSP. To focus on the purely domestic value-added one would need to combine firm and customs-level data.

10 Fig. 1 is drawn for the case where the linear slopes of α and x are equal but of opposite sign. In this case, at the intersection of x and α we also have the BVA-maximizing ROO $r^{*}$.

11 To ensure that the first order condition is a maximum, see the appendix.

12 The Lambert W function is the inverse function of $f\left(x\right)=xe^{x}$.

13 Note that $t_{xpt}$ are bilateral preferential tariffs, and $T_{pt}$ is the MFN tariff.

14 Let us rewrite $r^{*}$ as a function of the parameter estimated under each of the four functional form assumptions, $r^{*}=f\left(\theta \right)$, where θ is the true parameter in each of the four cases (i.e., $\gamma^{r}$, $\delta^{r}$, or $\psi^{r}$). A first-order Taylor series approximation of $r^{*}$ is given by $r^{*}\approx f\left(\hat{\theta }\right)+f^{\prime }\left(\hat{\theta }\right)\left(\hat{\theta }-\theta \right)$, where $\hat{\theta }$ is the point estimate of each parameter. Taking the variance on both sides of the equality, recalling that the variance of the mean and of the true parameter are zero, yields $var\left(r^{*}\right)={\left(f^{\prime }\left(\hat{\theta }\right)\right)}^{2}var\left(\theta \right)$. Taking the square root on both sides gives the expressions in equations (29), (30), (31). For the standard error of $r^{*}$ in equation (28), we use the implicit function theorem to obtain $f^{\prime }\left(\hat{\zeta^{r}}\right)$, as we do not have a closed-form solution for $r^{*}$.

15 There is, however, a growing literature on preference utilization. For a recent example, see [1] who show a negative correlation between preference utilization and the restrictiveness of EU rules of origin faced by UK exporters.

16 The average rule of origin requirement in the first year of our sample was 58%. In the last year of the sample the average rule of origin requirement was 34%.

17 As can be seen from the descriptive statistics in Table 1, the average rule of origin tends to be significantly lower for EBA's beneficiaries.

18 In 2019 the highest value-added requirement in the GSP regime was 60% for imports of textile products (HS630790), and the lowest was 30% for vehicles (HS870340).

19 For data availability, see https://www.trademap.org/stDataAvailability.aspx.

20 See Ref. [11] for a description of the relative importance of different ROO criteria in preferential agreements. The value-added criteria is used as one of the potential criteria to establish origin in at least 40% of tariff lines.
